# B cell lymphoma in hiv transgenic mice

**DOI:** 10.1186/1742-4690-10-92

**Published:** 2013-08-28

**Authors:** Sabrina Curreli, Selvi Krishnan, Marvin Reitz, Yanto Lunardi-Iskandar, Mark K Lafferty, Alfredo Garzino-Demo, Davide Zella, Robert C Gallo, Joseph Bryant

**Affiliations:** 1 Institute of Human Virology, University of Maryland School of Medicine, Baltimore, MD 21201, USA; 2 Department of Medicine, University of Maryland School of Medicine, Baltimore, MD 21201, USA; 3 Biochemistry and Molecular Biology, University of Maryland School of Medicine, Baltimore, MD 21201, USA; 4 Microbiology and Immunology, University of Maryland School of Medicine, Baltimore, MD 21201, USA

**Keywords:** B lymphoma, HIV-1, Transgenic mice

## Abstract

**Background:**

Human Immunodeficiency Virus Type I (HIV-1) infection is associated with a high incidence of B-cell lymphomas. The role of HIV in these lymphomas is unclear and currently there are no valid in *vivo* models for better understanding HIV-related lymphomagenesis. Transgenic (Tg) 26 mice have a 7.4-kb pNL4-3 HIV-1 provirus lacking a 3.1-kb sequence encompassing parts of the *gag*-*pol* region. Approximately 15% of these HIV Tg mice spontaneously develop lymphoma with hallmark pre-diagnostic markers including skin lesions, diffuse lymphadenopathy and an increase in pro-inflammatory serum cytokines. Here we describe the phenotypic and molecular characteristics of the B cell leukemia/lymphoma in the Tg mice.

**Results:**

The transformed B cell population consists of CD19^+^pre-BCR^+^CD127^+^CD43^+^CD93^+^ precursor B cells. The tumor cells are clonal and characterized by an increased expression of several cellular oncogenes. Expression of B cell-stimulatory cytokines IL-1β, IL-6, IL-10, IL-12_p40_, IL-13 and TNFα and HIV proteins p17, gp120 and nef were elevated in the Tg mice with lymphoma.

**Conclusions:**

Increased expression of HIV proteins and the B-cell stimulatory factors is consistent with the interpretation that one or more of these factors play a role in lymphoma development. The lymphomas share many similarities with those occurring in HIV/AIDS^+^ patients and may provide a valuable model for understanding AIDS-related lymphomagenesis and elucidating the role played by HIV-1.

## Background

Human Immunodeficiency Virus Type I (HIV-1) infection is associated with an elevated incidence of B-cell non-Hodgkin’s lymphoma (NHL) and in recent years also with Hodgkin’s lymphoma [1]. NHL includes different pathologic subtypes; the most common are diffuse large B-cell lymphoma and Burkitt’s lymphoma [1]. Lymphoma risk is increased approximately 150- to 250-fold among HIV-infected patients compared with the general population [1-3]. HIV-associated lymphomas (HAL) are aggressive and involve extranodal sites [1]. HAL development is frequently preceded by persistent generalized lymphadenopathy, suggesting antigen-induced chronic B cell stimulation and a likely pathogenic link between B cell hyperplasia and AIDS-NHL [4-6]. Chronic B cell activation may drive proliferation of antigen-selected B cell clones that accumulate genetic lesions and ultimately undergo neoplastic transformation. Indeed, elevated serum levels of several B cell stimulatory factors, including cytokines [7] and other soluble proteins [7-9], occur before the diagnosis of AIDS-associated lymphoma. In addition, continued antigenic stimulation of B cells by HIV [10-12] or other infectious agents such as Epstein-Barr virus [13] or human herpesvirus 8 (HHV-8) [14] seems to be critical for lymphomagenesis.

Different mouse lines transgenic for HIV-1 have been generated [15-18] and have been useful for modeling AIDS-like pathologies [19]. One of these, the HIV-1 Tg mouse line Tg 26, has a pNL4-3 HIV-1 proviral transgene lacking parts of the *gag*-*pol* region [20] and has been extensively used to model HIV-induced pathologies [20-23]. Here we report a phenotypic and molecular characterization of B cell tumors that develop in Tg26 mice. Similar to human HAL, Tg lymphomas are preceded by diffuse lymphadenopathy and increased pro-inflammatory serum cytokines. The transformed B cell population consists of CD19^+^pre-BCR^+^CD127^+^CD43^+^CD93^+^ precursor B cells and are clonal.

Murine models for human AIDS-related B cell lymphomas have been lacking. Hence, Tg26 mice may represent an important tool for understanding the role of HIV-1 in lymphomagenesis.

## Results

### HIV Tg mice developing lymphoma have abnormal lymphoid phenotypes

**Figure 1:**
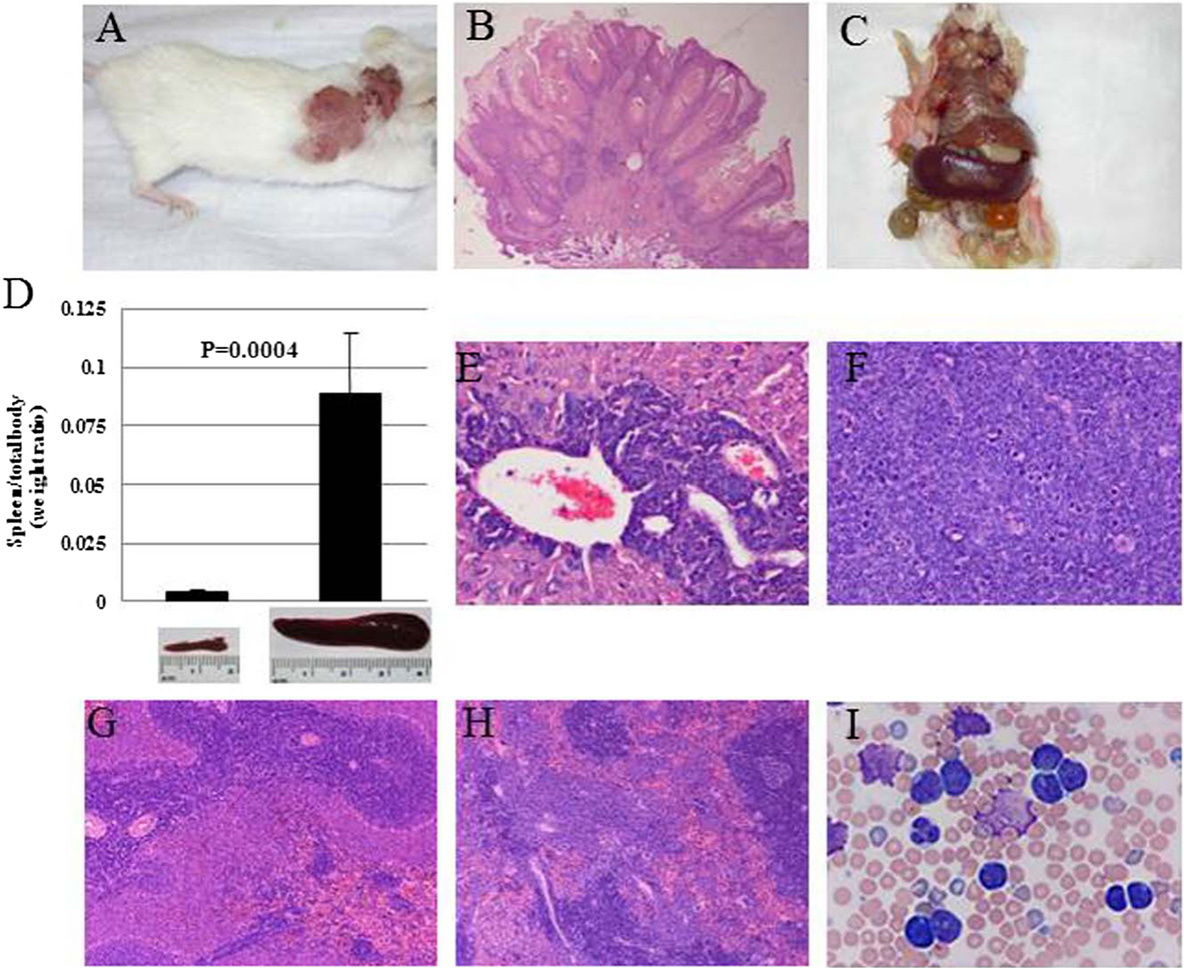
**Abnormal lymphoid phenotype in HIV Tg mice. (A-B)** Skin lesions in HIV-Tg mice. **(A)** Fungating lesions in the skin; **(B)** Histology shows a papilloma (low power). C-F Multi-organ involvement by lymphoma in the HIV-Tg mice. **(C)** Hepatosplenomegaly and lymphadenopathy (gross). **(D)** Spleen weight/total body weight ratio in HIV Tg mice compared to control wild type FVB/N. 10 HIV Tg and 10 control animals were analyzed. The spleen weight/body weight ratios were approximately 23 times greater than that of wild type (WT) mice (p=0.0004). **(E)** Portal infiltration by lymphoma cells in the liver (H&E; x400); **(F)** Lymphomatous infiltration of the lymph node (H&E; x400). G-H Comparison of control and HIV-Tg mouse spleen. **(G)** Normal splenic architecture in control mice (H&E; x400); **(H)** Disorganized splenic architecture with atypical lymphoid infiltration and numerous hyperplastic megakaryocytes (H&E; x400). **(I)** Wright-Giemsa staining of peripheral blood smears. The staining shows circulating lymphoblasts with scant basophilic cytoplasm, high nuclei to cytoplasm ratio, round to irregular nuclei, slightly clumped chromatin, and inconspicuous nucleoli. Small cytoplasmic and nuclear vacuoles were occasionally seen.

HIV Tg26 heterozygous mice share a common phenotype characterized by cataracts, cutaneous papillomas (Figure 1A, 1B) and renal disease [20-23]. The percentage of heterozygous Tg mice with skin lesions increased with age and varied from 18% to 59%, as previously reported [22]. By 8–12 months of age approximately 15% of the mice with cutaneous papillomas (15/100) developed splenomegaly, lymphadenopathy and extra-nodal involvement of liver, gastrointestinal tract and central nervous system (Figure 1C, D). H&E staining of lymphoid organs liver (Figure 1E), lymph node (Figure 1F) and spleen (Figure 1H) showed all with atypical lymphomatous infiltration. Spleen sections (Figure 1G, H) showed a disorganized spleen architecture with loss of germinal centers and atypical lymphoid infiltration. Peripheral blood smears (Figure 1I) displayed circulating lymphoblasts.

### Expression of HIV mRNA and proteins in Tg mice with splenomegaly

To measure HIV Tg expression, spleen and lymph node sections from Tg26 mice at different stages of disease were analyzed for HIV-1 unspliced (US), single spliced (SS) and multiply spliced (MS) transcripts [24]. Viral RNA was quantified in HIV Tg mice at different stages of splenomegaly by semiquantitative real-time RT-PCR and was expressed as fold increase relative to the levels expressed in Tg26 mice with no signs of disease. Signs of disease progression in Tg mice with skin papillomas were defined based on clinical signs (e.g. abdominal enlargement and ragged fur) and by analyses of T/B cell ratios in peripheral blood (Table 1).

**Table 1:** HIV gene expression in HIV Tg mice

	**SPLEEN**				**LYMPH NODE**				**LYMPH NODE/SPLEEN**		
**Mouse number**	**T/B ratio**	**HIV genes**			**T/B ratio**	**HIV genes**			**HIV genes**		
		**MS**	**SS**	**US**		**MS**	**SS**	**US**	**MS**	**SS**	**US**
**M5**	23/65	-	-	-	86/12	-	-	-	-	-	-
**M15**	25/74	0.56	0.26	0.78	nd	2.56	1.4	1.48	4.57	5.38	1.90
**M22**	5/69	0.18	0.0	0.45	70/30	0.87	6.28	1.24	4.83	6.28	2.76
**M21**	3/60	0.20	0.48	0.68	30/70	0.7	0.91	1.11	3.50	1.90	1.63
**M7**	4/96	2.11	2.7	0.62	25/75	1.62	3.46	5.00	0.77	1.28	8.06
**M16**	5/83	0.66	1.53	0.87	12/78	0.87	2.67	1.20	1.32	1.75	1.38
**M20**	2/58	0.04	0.1	0.09	1/92	18.9	20	5.55	˃100	>100	61.67
**M13**	1/83	1.36	1.37	0.43	5/95	1.9	2.44	7.81	1.40	1.78	18.16
**M2**	4/86	0.37	0.8	2.36	11/84	0.17	1.13	1.29	0.46	1.41	0.55
**M11**	2/90	1.64	5.12	0.96	1/92	0.77	1.78	1.14	0.47	0.35	1.19

Viral RNA was quantified in HIV Tg mice at different stages of splenomegaly by semiquantitative real-time RT-PCR and was expressed as fold increase relative to the levels expressed in Tg 26 mice with no signs of disease. The lefts column indicates the numbers assigned to the mice. Both spleen (on the left) and lymph node (on the center) were analyzed for each mouse. The table shows T/B ratio and semiquantitative real-time RT-PCR of HIV MS (multiply spliced), SS (single spliced) and US (unspliced) RNAs. The ratio of the RNA levels in the lymph node versus that in the spleen of the same mouse is shown on the extreme right.

There were considerable differences in the levels of HIV genes expressed in the spleen and the levels expressed in lymph nodes from the same mouse (Table 1). Minimal changes in HIV gene expression in the spleen occurred with disease development (0.74±0.23-fold increase in MS RNAs over controls, 1.26±1.6-fold and 0.8±0.26-fold increase in SS and US RNAs over controls, respectively). In contrast, HIV gene expression in lymph nodes, although still highly variable, was increased relative to controls (2.87±1.8-fold increase in MS RNAs over controls, 4.09±1.8-fold and 2.87±0.95-fold increase in SS and US RNAs over controls, respectively).

However in mice with T/B ratio of 25/75 and lower, difference in viral RNA levels in spleen and in lymph nodes from the same mouse was less evident. In particular, expression of HIV MS and SS RNAs in the lymph node was reduced and nearly similar to the expression level in spleen, while expression of US RNAs did not correlate with T/B ratio (Table 1). This reduction in the levels of HIV genes expressed in lymph nodes is probably due to B cell infiltration in the lymph node during disease progression.

**Figure 2:**
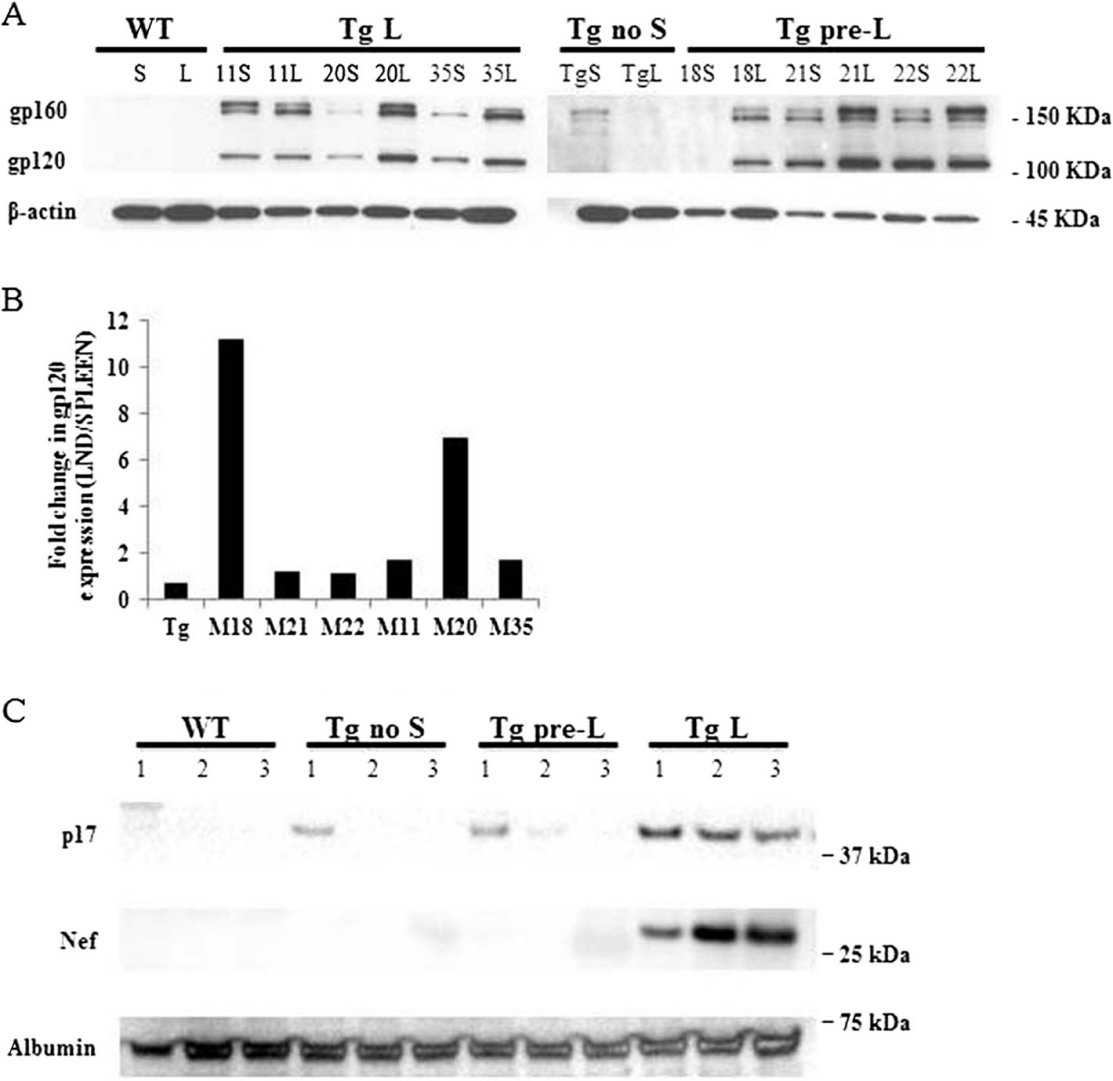
**HIV proteins expression in HIV Tg mice. (A)** gp120 expression in HIV Tg mice. Protein lysates from mice spleen and lymph node were separated by SDS-PAGE and analyzed by western blot using anti-gp120 antibody as described under “Experimental Procedures”. The mice analyzed are indicated on the top of the figure: WT is the mouse control FVB/N, Tg is an HIV Tg mice without skin lesions, mice number 18, 21, and 22 represent mice at the pre-lymphadenopathy stage while mice number 11, 20 and 35 represent mice at a late stage of lymphadenopathy. The numbers in the right represent the protein molecular weight in KDa. **(B)** Histogram showing the fold changes in gp120 expression in the lymph node versus the spleen from the same mouse. The relative intensity of gp120 was quantified by densitometry and normalized with the beta actin. **(C)** Western blot for p17 and nef proteins expression in HIV Tg mice using anti-p17 antibody and HIV-1 Nef antiserum as described under “Experimental Procedures”. The mice analyzed are indicated on the top of the figure: WT are mice wild type control FVB/N, Tg are HIV Tg mice without skin lesions, Tg pre-L are mice at pre-lymphadenopathy stage and Tg-L are mice at the last stage of lymphadenopathy. The protein molecular weights are represented in the right of the figure.

In support of viral RNA expression data, we performed Western blots to measure HIV-1 gp120 protein in Tg mice at different stages of disease (Figure 2A, B). Expression of gp120 was higher in lymph node versus spleen from the same mouse. To determine if HIV-1 genes are differentially expressed at the protein level in Tg mice at different stages of lymphadenopathy, proteins from splenocytes isolated from 3 WT, asymptomatic Tg mice without skin lesions (Tg no S), Tg mice at early stages of lymphadenopathy (Tg pre-L), and Tg mice at late stages of lymphadenopathy (Tg L) were subjected to SDS-PAGE and immunoblotting with anti-HIV antibodies specific for p17 and nef (Figure 2C). We found that Tg mice at late stages of lymphadenopathy express elevated levels of HIV proteins known to interact with B cells (p17, gp120, and nef) [10,25,26] compared to asymptomatic Tg mice without skin lesions (Figure 2). Further, Tg mice at early stages of lymphadenopathy had elevated levels of p17 and gp120 but not nef supporting a possible role of p17 and gp120 in lymphomagenesis.

### Elevated expression of pro-inflammatory cytokines in Tg mice

**Figure 3:**
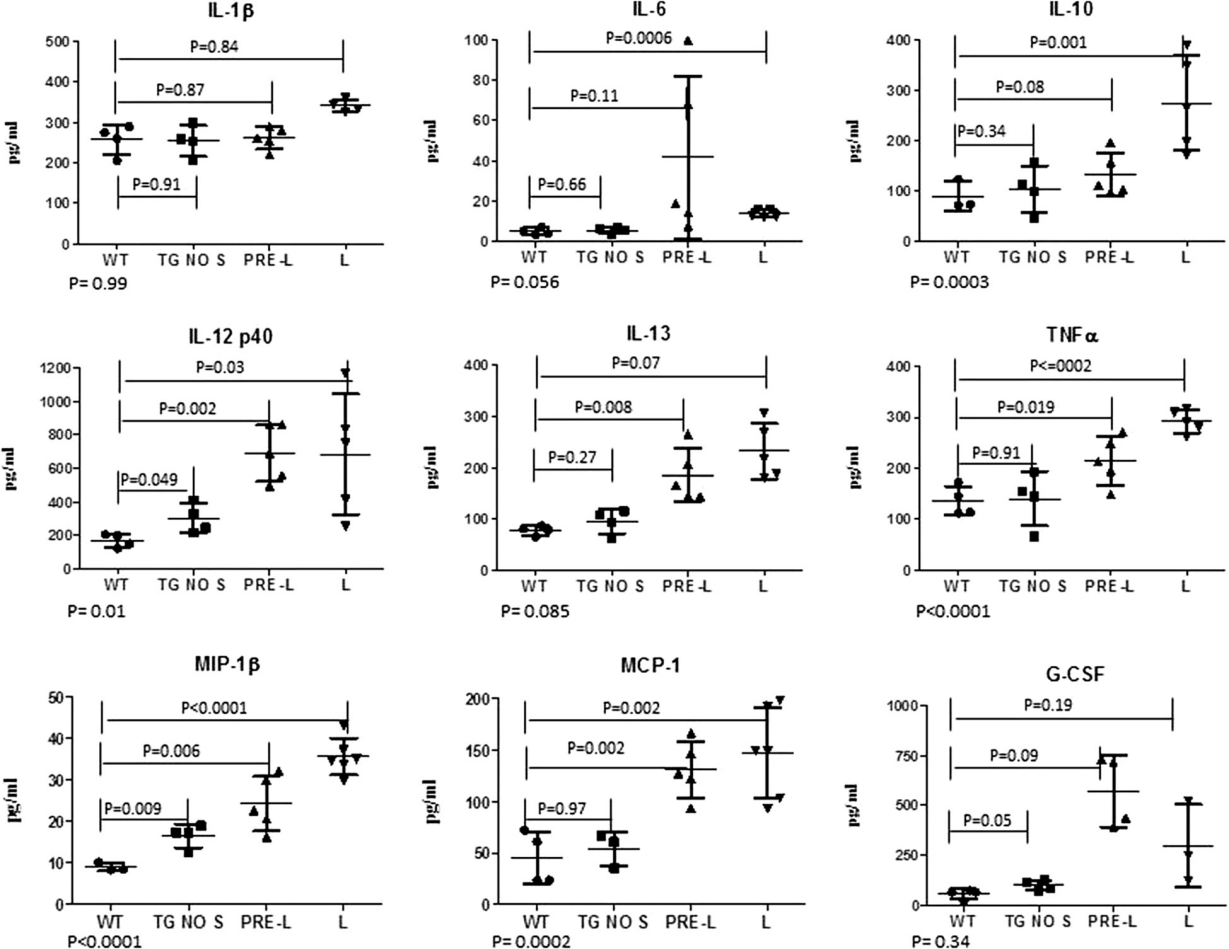
**Cytokines and chemokines levels in the plasma of HIV Tg mice.** Cytokine and chemokines production was compared among four different groups: (i) FVB/N WT control (WT) (solid circles), (ii) asymptomatic Tg mice without skin lesions (Tg no S) (solid squares), (iii) Tg mice at early stages of lymphadenopathy (pre-L) (solid triangles) and (iv) Tg mice at the late stage of lymphadenopathy (L) (inverted solid triangles). Plasma samples from each group were collected and analyzed in triplicate. Bars denote the standard deviation. The p-values are represented as lines in the figures and were calculated as unequal variance t-test between Tg no S, pre-L and L relative to the WT. The p values for anova are at the bottom of each figure.

Lymphoma in HIV^+^ patients is associated with immune deregulation [9] and abnormal expression of certain cytokines and chemokines [27,28] are considered biomarkers for lymphoma development. To determine if disease progression in HIV Tg mice is associated with a similar cytokine signature, we measured cytokine levels in the plasma of Tg mice at different stages of lymphadenopathy. Expression of IL-1β, IL-6, IL-10, IL-12_p40,_ IL-13, TNFα and chemokines MIP-1β MCP-1 and G-CSF (Figure 3) by asymptomatic Tg no S was greater than that of WT controls, although only for IL-12_p40_ (*P*=0.049)_,_ MIP-1β (*P=*0.009), and G-CSF (*P*=0.05) were the changes significant. Differences were more evident, however, in levels of IL-12_p40_ (*P*=0.002), IL-13 (*P*=0.008), TNFα (*P=*0.019) and chemokines MIP-1β (*P=*0.006), MCP-1 (*P*=0.002) (but not IL-1β, IL-6, IL-10 and G-CSF) when comparing Tg pre-L to WT controls (Figure 3). Similarly, IL-6 (*P*=0.0006), IL-10 (*P*=0.001), IL-12_p40_ (*P*=0.03), TNFα (*P≤*0.002) and chemokines MIP-1β (*P<*0.0001) and MCP-1 (*P*=0.002) (but not IL-1β, IL-13 and G-CSF) were significantly elevated in Tg mice at late stages of lymphadenopathy (Tg L) relative to WT controls. Collectively the data show significant and progressive increases in plasma IL-1β, IL-6, IL-10, IL-12_p40_, IL-13 and TNFα during progression to splenomegaly, suggesting that, as in HIV patients developing lymphoma [27,28], these changes are prognostic for the development of lymphadenopathy and splenomegaly in HIV Tg mice. We also observed increases in expression of MIP-1β, MCP-1 and G-CSF; however, no association of these chemokines with HIV-related lymphomas has been reported.

### Loss of peripheral B cell lineage and expansion of precursor B cell populations in HIV Tg mice with splenomegaly

**Figure 4:**
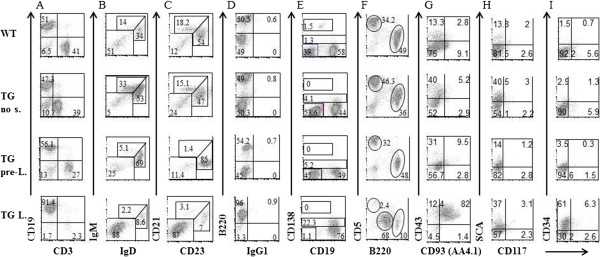
**Splenic peripheral B cell population in HIV Tg26 mice.** Flow analysis was performed in splenocytes from wild type mouse FVBN (WT), Tg26 without skin lesions (Tg no S), Tg26 at early stage of splenomegaly (Tg pre-L) and a Tg at advanced stage of splenomegaly (Tg L). Splenic B cells comprised two major populations at side/forward scattered-high (SSC/FSC_hi_), corresponding to non-activated and activated populations (Figure 3 shows only the activated population). Starting from the left side, flow cytometry analysis of splenic B cell population in HIV Tg26 mice displayed: **(A)** decrease of T versus B cell ratio, as shown by CD3 and CD19 staining; **(B)** decrease of immature (B220^+^IgD^-^IgM^+^) and mature (B220^+^IgD^+^IgM^-^) B cells; **(C)** decrease in follicular (B220^+^CD21^-^CD23^+^) and marginal zone (B220^+^CD21^+^CD23^-^) B cells; **(D)** no change in IgG1-expressing B cells (B220^+^IgG1^+^); **(E)** reduction in plasma cells (CD19^+^CD138^+^); **(F)** decrease in B1B cells (B220^+^CD5^+^); **(G)** increase of precursors B cells (B220^+^CD43^+^CD93^+^); **(H)** increase of B220^+^Sca^+^ B cells; undetectable c-kit and **(I)** increase of B220^+^CD34^+^ B cells.

To better characterize the splenomegaly, splenic B cell populations from Tg mice at different stages of disease progression were analyzed by flow cytometry (Figure 4). Consistent with the splenomegaly, splenic B/T cell ratios were increased in mice progressing toward splenomegaly. We observed a loss of most of the peripheral B cell lineage, including immature, mature, follicular, marginal zone B cells, plasma cells, and B1b cell population (Figure 4). HIV Tg26 mice during the last stages of splenomegaly had an expanded immature B cell population consisting of CD19^+^B220^+^IgM^low^IgD^-^CD21^low^CD23^-^CD138^+^CD5^+^ cell. Staining for precursor B cell markers [29] revealed that the majority of B cell populations circulating in peripheral blood as well in bone marrow and spleen during the last stages of splenomegaly consisted of B220^+^CD19^+^CD43^+^CD93^+^CD127^+^ precursor B cells (Figure 4G, Additional file 1: Figure S1 and Additional file 1: Table S1). These cells expressed mRNA for rag 1 and rag 2 enzymes (data not shown), suggesting that the cell population invading the spleen derived from a B cell precursor lying between the pro-B fraction B/C and the pre-B fraction D based on the Hardy’s classification [29]. Finally, analysis for expression of the hematopoietic stem cell markers CD34, c-kit and Sca-1 showed that while c-kit expression remained mostly undetectable, Sca-1 and CD34 were expressed at high levels in mice with splenomegaly (Figure 4H, I).

### B cell clonality in HIV Tg mice with splenomegaly

In order to evaluate B cell clonality we used a PCR-based method with primers specific for the D-J segment of IgH [30]. DNA analysis of the spleen from HIV Tg mice at different stage of disease progression shows that both mice progressing to splenomegaly (Additional file 1: Figure S2A) and mice at the last stage of splenomegaly (Additional file 1: Figure S2B) were polyclonal. However while mice progressing to splenomegaly were comparable to the WT control, some of the mice at the last stage of splenomegaly showed preferential amplification of one of the D-JH family of Ig variable genes, such as D-JH3 for mice n.20, 28, 34, 37 and 11, or D-JH1 for mouse n.16 (Additional file 1: Figure S2B). This suggests the emergence of a clonal B cell population occurring with advanced disease.

### Oncogene expression in HIV Tg mice

We used real-time semiquantitative RT-PCR to measure expression of different oncogenes associated with B cell lymphomas [31-34] in Tg mice (Additional file 1: Table S2). Based on oncogene expression patterns in the spleen and the lymph node (Table 2), mice at different stages of disease were categorized into three groups (Additional file 1: Figure S3 and S4). Group A included mice where oncogene expression was ≤ 5 times greater than that of WT controls. Group B included mice between a >5 to ≤ 10 fold increase in oncogene expression compared to that of controls. For group C, the fold increase was > 10. Small changes in the expression of multiple oncogenes were observed in group A, while mice in group B and to a greater extent in group C, in addition to variable expression of multiple oncogenes, had a dominant oncogene that was highly expressed, suggesting that by this stage of disease genetic changes had probably occurred. Of note, data with the spleen (Additional file 1: Figure S3) are in agreement with the clonality assay shown in Additional file 1: Figure S2. Indeed in mouse n.11 from group B and in mice n.28 and n.16 from group C with a greater than 10 fold increase in relative expression of an oncogene, there was also preferential amplification of one of the D-JH segments, suggesting a shift in these mice of B cell populations towards oligoclonality.

**Table 2:** Oncogenes expression in HIV Tg mice

		**T/B**	**BCL2**	**BCL3**	**BCL6**	**C-REL**	**MUM1**	**C-MYC**	**CYC D1**	**SYK**	**K-RAS**	**ABL**
M4	S	77(2/58)	0.09	2.51	8.75	0.14	0.02	2.25	14.13	0	0.79	1.95
	L											
M9	S	nd	1.00	0.94	1.32	0.39	0.69	0.58	0.81	nd	1.59	nd
	L											
M24	S	nd	3.7	2.55	4.9	6.15	0.71	1.99	3.65	2.12	1.98	nd
	L	nd	2.68	1.00	0.42	2.91	0.35	0.04	0.54	0.33	1.63	1.07
M25	S	32(47/54)	4.99	4.98	8.88	4.42	0.75	0.89	2.85	0.60	0.80	0.76
	L	nd	3.13	0.06	1.00	7.14	0.64	0.64	0.26	3.88	3.16	1.48
M1	S	26(31/58)	2.77	1.14	1.58	1.59	1.14	0.23	1.68	0.02	1.44	1.24
	L	71(77/23)	0.9	2.5	1.06	0.78	3.49	0.43	0.59	2.56	0.63	nd
M3	S	25(16/77)	2.52	3.67	2.38	6.62	0.67	3.6	1.88	7.7	2.31	nd
	L	nd										
M15	S	23(25/74)	0.28	0.61	1.02	0.12	0.19	0.93	0.70	1.56	2.31	1.59
	L	nd	2.93	0.32	1.80	6.95	0.4	0.77	0.28	1.98	2.83	1.12
M22	S	61(5/69)	0.32	1.39	0.71	0.27	0.95	0.32	0.88	1.34	0.18	5.91
	L	93(70/30)	3.33	0.68	0.19	3.33	0.94	0.52	0.25	0.87	5.33	8.39
M21	S	60(3/60)	1.98	0.96	2.64	1.03	2.56	1.92	3.92	2.46	0.44	1.91
	L	80(30/70)	0.92	0.79	0.19	2.45	1.28	0.07	0.65	3.66	3.93	2.25
M2	S	20(4//86)	0.46	0.20	1.09	0.07	4.21	2.52	8.75	0.06	2.29	2.67
	L	25(25/75)	0.58	0.4	1.12	1.07	1.7	3.23	0.47	3.18	1.5	5.01
M7	S	80(4/96)	0.21	0.53	0.22	0.46	5.1	0.60	0.41	2.32	2.98	2.27
	L	54(27/75)	0.43	0.39	0.41	2.32	5.78	2.56	0.8	8.75	9.39	9.19
M11	S	85(2/90)	1.74	3.27	5.17	8.23	7.02	2.74	1.23	8.69	2.89	3.84
	L	88(1/92)	0.04	2.69	0.2	2.83	5.6	0.78	0.13	4.6	1.89	3.41
M13	S	40(2/77)	0.44	1.57	1.94	2.69	2.99	1.78	0.61	3.45	2.77	0.17
	L	40(5/95)	2.29	0.70	0.24	9.19	4.74	0.30	0.56	21.45	8.96	6.4
M16	S	35(5/83)	3.50	0.80	3.68	13.48	2.07	1.81	5.13	8.4	19.45	nd
	L	53(15/85)	0.73	1.49	0.28	4.52	0.95	3.61	4.91	2.53	16.76	8.23
M20	S	77(2/58)	0.21	0.86	0.23	0.23	0.46	0.68	0.72	1.41	0.25	0.93
	L	75(1/99)	0.06	0.22	0.07	0.89	2.7	0.96	0.15	4.64	2.01	5.31
M28	S	55(2/90)	0.38	0.72	0.69	1.03	5.96	15.38	0.41	1.51	6.28	0.11
	L	nd	0.42	0.21	0.18	0.55	nd	5.54	0.28	1.98	2.1	3.35

Total RNA was isolated from spleen and lymph node of mice at different stage of splenomegaly and was analyzed using primers specific for oncogenes associated to lymphoma (Additional file 1: Table S2) by SYBR Green semiquantitative real-time RT-PCR as described in material and methods. The fold change in each oncogene mRNA compared to the wild type FVBN control is shown relative to the change in the expression of β-actin that was measured as an internal control. The expression of each oncogene in the wild type FVBN control was set to 1. Each mouse is designated with M followed by a number (first column). For each mouse the first line represents the spleen (S), second line represents lymph nodes (L).T/B ratio is indicated in parenthesis and is calculated on total activated cells.

Although there was no consistent elevation of expression of specific oncogenes, different oncogenes were upregulated in different mice progressing to splenomegaly. Among the analyzed oncogenes bcl6, syk, k-ras, c-rel, c-abl and cyclin D1 were variably increased, while bcl2 and bcl3 did not change greatly (Table 2). The aberrant expression of oncogenes in spleen and lymph node was often correlated with T/B cell ratios, both of which were related to disease progression. When oncogenes expressed in the spleen were compared with oncogenes expressed in the lymph node from the same mouse, common oncogenes were increased in mice n. 16, 28, 11, 22, 7 and 25 (Table 2). However, oncogenes that were overexpressed in spleen and lymph node were not the same in the majority of the mice analyzed, suggesting that stochastic events or different micro-environmental stimuli modulate oncogene expression in the two tissues.

### B cells from HIV Tg mice with splenomegaly are tumorigenic

Splenic B cells were isolated from a mouse at the last stage of splenomegaly and lymphadenopathy (mouse n.28 in Table 2). The CD3^-^ population from the mouse splenocytes was further separated into four fractions based on the B cell markers B220^+^, CD19^+^, CD93^+^ and CD117^+^. 10^6^ cells from each purified B cell population were injected intraperitoneal into NOD/SCID mice.

**Figure 5:**
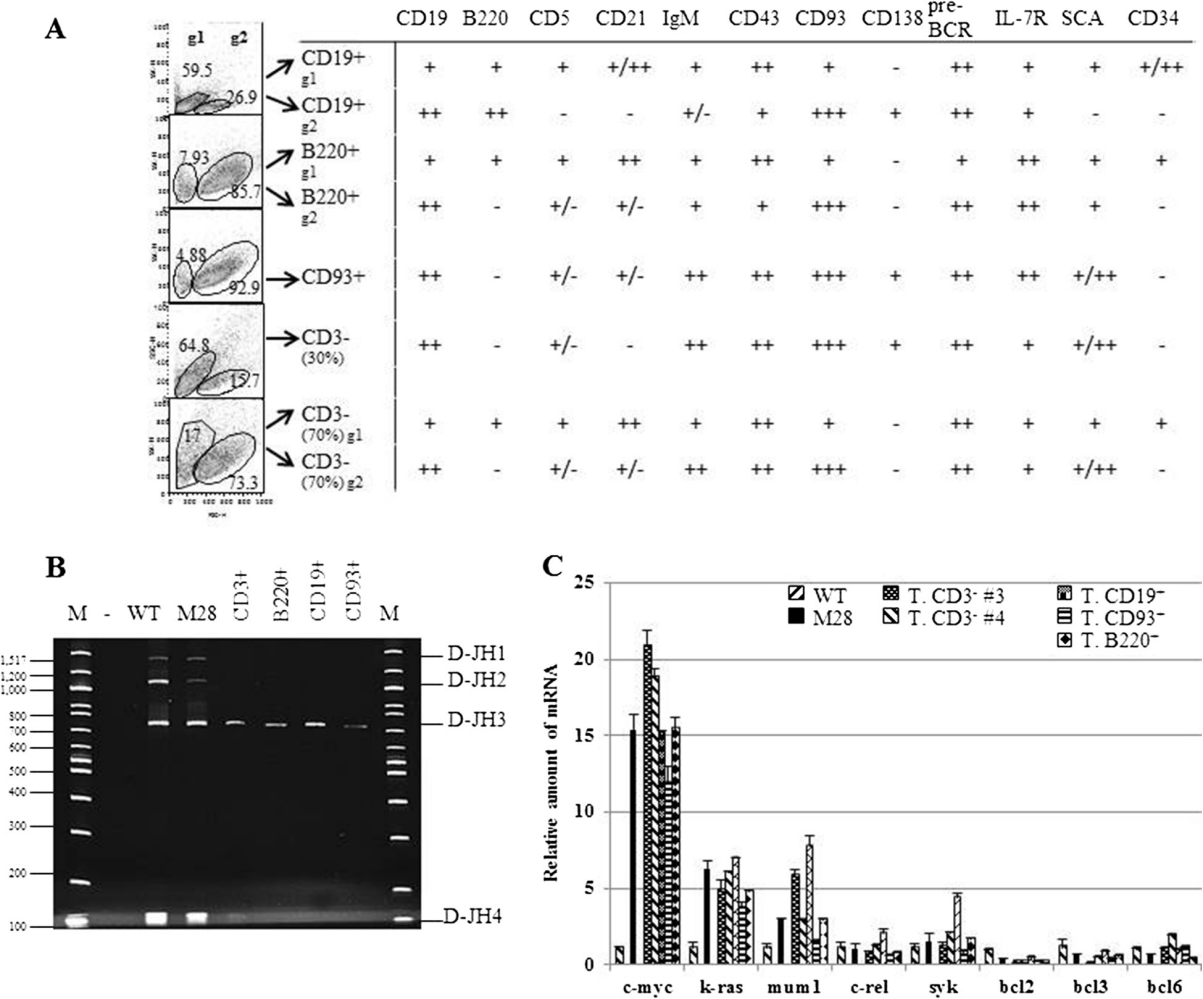
**Tumors characterization. (A)** Phenotypic analysis of extranodal tumors developed in NOD/SCID mice injected with splenocytes from HIV Tg mouse #28. Flow analysis was performed in tumors from mice from groups injected with CD19^+^, B220^+^, CD93^+^, and the control CD3^-^. Mice shown are representative from each group. Tumors were analyzed for the following cell markers consisting in: CD3, CD19, CD43, CD93, B220, IgM, CD21, CD5, pre-BCR, Sca1, CD127, CD138, and CD34. “g1”and “g2” indicate the not activated and activated gates, respectively. “+”, “++”, “+++” indicate peak fluorescence intensity one, two or three logs higher than isotype control, respectively; “-” indicates no change in peak fluorescence intensity compared to isotype control; “+/−” indicates peak fluorescence intensity less than one log higher than isotype control. Flow histograms of extranodal tumors are shown in Additional 1: Figure S7. **(B)** Analysis of D-J rearrangement of the IgH gene. Genomic DNAs from extranodal tumors developed in NOD/SCID injected with B220^+^, CD19^+^, CD93^+^ and CD3^-^ splenocytes purified from HIV Tg mouse n.28 were analyzed for D-J rearrangement of the IgH gene. Splenocytes from a wild type (WT) FVBN mouse were used as a control polyclonal B cells show four bands corresponding to D-JH1, D-JH2, D-JH3 and D-JH4, indicating that these four segments were rearranged, while tumors were monoclonal. The repertoire of Ig genes utilized in the monoclonal tumors indicated a preferential usage of the D-JH3 family of Ig H variable genes. **(C)** Molecular analysis of oncogenes expressed in extranodal tumors developed in NOD/SCID injected with B220^+^, CD19^+^, CD93^+^ and CD3^-^ splenocytes purified from HIV Tg mouse n.28. A single mouse representative for each group of mice injected is shown. Oncogenes analysis was performed by SYBR Green semiquantitative real-time RT-PCR as described in material and methods.

Of the mice injected with selected B cell populations, 40% of B220^+^ and CD19^+^ injected mice developed lymphoma, but only 20% of those injected with CD93^+^ cells developed lymphoma and all of those injected with CD117^+^ cells or CD3^+^ control cells remained healthy (Additional file 1: Figure S5). FACS analysis demonstrated infiltration of CD19^+^ cells into lymphoid organs (Additional file 1: Figure S6). Phenotypically, the tumors from the injected mice consisted of one or two main populations at SSC/FSC_hi_ (Figure 5A and Additional file 1: Figure S7). While mice injected with B220^+^ and CD19^+^, as well 70% of the total CD3^-^ injected mice developed tumors consisting of one population of non-activated and one population of activated B cells, tumors in CD93^+^ injected mice and in 30% of the total CD3^-^ injected mice included only one activated population. The activated tumorigenic population, commonly present in all tumors, was B220^+/−^CD19^+^CD43^+^CD93^+^pre-BCR^+^CD127^+^IgM^+^CD21^+/−^CD5^+/−^CD138^+^Sca1^+^ and CD34^-^, whereas the non-activated B cell population was B220^+^ CD19^+^CD43^+^CD93^+^pre-BCR^+^CD127^+^IgM^+^CD21^+^CD5^+^CD138^-^Sca1^+^ and CD34^+^. The B cell marker B220 was expressed only in the non-activated B cell population from all tumors, with the exception of the those generated from CD19^+^ injected mice, which were consistently B220^+^ (Figure 5A and Additional file 1: Figure S7). B cell markers CD21, CD5, CD138 and CD34 were variably expressed in the tumorigenic population and probably simply reflected the activation stage of the cells.

To determine whether the phenotypic differences in the secondary tumors reflect a difference in the clonal origin of the tumors, we evaluated B cell clonality. Whereas splenocytes from mouse n.28, from whom the tumorigenic B cells were derived, were polyclonal (Additional file 1: Figure S2B), the tumors that developed in recipient mice displayed a monoclonal IgH rearrangement (Figure 5B). These results suggests that only one of the multiple B cell clones detected by IgH rearrangements in the HIV Tg mouse n.28 produced the malignant B clones in all secondary tumors, while other B cell clones from this mouse, although immortalized were not (or at least were less) malignant.

We compared the oncogene expression pattern of HIV Tg mouse n.28 (Table 2) with the oncogenes expressed in the tumors developed in the NOD/SCID mice injected with B cells fractions from mouse n.28 (Figure 5C). Molecular analysis of the oncogenes expressed in the spleen from mouse n.28 showed increased expression of c-myc (15.38 ± 0.96), k-ras (6.28 ± 0.56) and mum1 (2.94 ± 0.1). In contrast, there was no significant increase in the expression of c-rel, syk, bcl2, bcl3 and bcl6. RNA from the tumors that developed in mice injected with B cell fractions B220^+^, CD19^+^, CD93^+^ and CD3^-^ controls expressed, similar to mouse n.28, elevated levels of c-myc, k-ras and mum1 oncogenes with a fold increase comparable to mouse n.28 (Figure 5C). These results demonstrate that the tumorigenic B cell population derived from mouse n.28 preserved its molecular signature in the recipient NOD/SCID mice and suggest a role for c-myc in lymphomagenesis.

### HIV Tg mice developing lymphoma do not express *v-abl*

A similar pattern of pre-B cell transformation occurs following infection with Abelson murine leukemia virus (Ab-MLV) which encodes the *v-Abl* tyrosine kinase gene [35,36]. In order to exclude the possibility that the pre-B cell transformation in HIV Tg mice was due to infection with Ab-MLV, we tested DNA and cDNA from the spleen of mice with lymphoma for the presence of *v-abl*. Four different sets of primers were tested since *v-Abl* is expressed in different forms [35]. We used primers 124F/R, 310F/R, 382F/R and 4664F/R (Additional file 1: Table S3) that target diverse regions of the viral protein but do not recognize *c-abl*. The macrophage cell line Raw 264.7 cDNA [37] was the positive control. None of the tested primers detected *v-abl* mRNA (data not shown). However, expression of the cellular oncogene *c-abl* was variably modulated in HIV Tg mice with lymphoma (Table 2).

## Discussion

In this report we characterize the phenotypic and molecular features of B cell leukemia/lymphomas that develop in Tg26 mice HIV-1 Tg mice. About 15% of Tg mice develop B cell lymphomas with a constellation of pre-diagnostic markers similar to those of B cell lymphoma in HIV patients, thus providing the first animal model for elucidating the mechanisms by which HIV infection leads to AIDS-related B cell lymphomas.

The lymphomagenic role of HIV-1 is not well understood, although HIV-induced chronic B cell stimulation appears to be critical [4-6]. Numerous studies suggest roles for several HIV proteins, including Tat [11], Nef [26], Env gp120 [10], and Gag p17 [25], in mediating B cell hyper-proliferation [4-6] that precedes lymphoma in HIV patients. HIV-1 induces B cell-stimulatory cytokines including IL-6 and IL-10, which are pre-diagnostic for HAL [7,27,28]. Similarly to the clinical data, Tg26 mice with leukemia/lymphoma displayed: (i) splenomegaly, lymphadenopathy and extra nodal enlargements in the liver and the gastrointestinal tract (Figure 1); (ii) increased lymph node expression of HIV-1 genes (Table 1), increased expression of HIV proteins p17, gp120 and nef in mice developing lymphoma (Figure 2); and (iii) significant increases in serum cytokines including IL-6, IL-10, TNF-α, IL-12 and IL-13 (Figure 3). HIV-1 structural proteins gp120 and p17 persist in patients under high active antiretroviral therapy [38], and the increased expression of p17, gp120 and nef in Tg 26 mice developing lymphoma suggest a lymphomagenic role of these proteins.

Loss of peripheral B cell subpopulations and infiltration of lymphoid organs by polyclonal B cells was a hallmark of HIV-1 Tg mice progressing to lymphoma. The infiltrating B cell population retained a phenotype with specific markers of precursor B cells (B220^+^CD19^+^CD43^+^CD93^+^CD138^+^CD127^+^) [29] and expressed markers of immature B cells (IgM^low^IgD^-^CD21^low^CD23^-^) [39] and common lymphoid precursors (Sca1^+^c-Kit^-^CD34^+^) [40] (Figure 4 and Additional file 1: Figure S1 and Additional file 1: Table S1).

B220^+^, CD19^+^ and CD93^+^, but not c-kit^+^ splenocytes isolated from an HIV Tg mouse in the last stage of splenomegaly and lymphadenopathy induced tumors in NOD/SCID mice. The tumorigenic pre-B cell population was phenotypically similar to splenocytes from HIV Tg mice with splenomegaly and consisted of B220^+/−^CD19^+^CD43^+^CD93^+^pre-BCR^+^CD127^+^CD138^+^IgM^+^CD21^+/−^CD5^+/−^Sca1^+^ and CD34^+/−^, with B cell markers B220, CD21, CD5, CD138 and CD34 variably expressed and probably linked to the activation stage of the cells (Figure 5A and Additional file 1: Figure S7).

Transplant tumors were monoclonal (Figure 5B), in contrast to primary tumors, which were polyclonal (Additional file 1: Figure S2). Therefore we assume that both lymphoproliferation and ultimately full neoplastic transformation occur in B cells from HIV-1 Tg mice and that the malignant lymphomas arise from among the multiple B cell clones detected by PCR clonality assays. The presence of pre-B cells in the blood and bone marrow of Tg mice developing lymphoma and infiltrating the lymphoma itself define this malignancy as precursor B cell leukemia/lymphoma.

We speculate that lymphomagenesis in Tg26 mice is due to a paracrine effect of viral or virally induced cellular proteins acting on a stem-like hematopoietic cell belonging to a common lymphoid precursor or a pre-B cell. In this scenario HIV would perturb B cell differentiation, with consequent immortalization and transformation of cells at the B cell precursor stage. Indeed, the regulation of proliferation of precursor B cells is an essential checkpoint in B cell development and therefore is particularly prone to transformation. The differentiation of pre-B cells is dependent upon the regulatory interplay between IL-7 receptor and pre-BCR signaling [41,42], as well the activity of B cell transcription factors [43]. Active pre-BCR induces cell division, recombination of the immunoglobulin light chain and differentiation of pre-B cells in immature B cells expressing BCR [29]. However, defects in pre-BCR assembly and signaling may interfere with B cell development or may cause uncontrolled proliferation and genomic instability, resulting in immunodeficiency or tumor development. It is possible that one or more HIV proteins interfere with differentiation of pre-B cells and delineation of the early stages of B cell development in the Tg26 mice help establish a role for HIV in lymphoma development. Nevertheless these studies will need to exclude a role in lymphomagenesis for viral proteins expressed within the B cell population of the Tg model, suggesting the need for an updated mouse model expressing the Tg only in naturally HIV permissive cells.

Deregulated expression of one or more cellular oncogenes is usually associated with cancer, including B cell lymphomas [44,45] and leukemia [46]. ARL are characterized by the presence of recurrent oncogene translocations [32,33], or by inactivating mutations and deletions of the *p53* tumor suppressor gene [47]. Molecular analysis of oncogene expression in Tg26 mice shows an increased expression of multiple oncogenes with the dominant expression of single oncogenes in mice progressing to lymphoma. Oncogenes bcl6, syk, k-ras, c-myc and cyclin D1 were variably increased, while bcl2 and bcl3 did not change considerably. The aberrant expression of oncogenes in tg mice was often correlated with T/B cell ratios, both of which were related to disease progression.

Early studies of the HIV Tg26 line indicated that the transgene is integrated in chromosome 8 in multiple copies [20]. Analysis of the integration sites will obviously be important for understanding whether and how the transgene may be causing genome instability compared to the WT mouse. These studies are planned for the near future.

Another potential role of HIV in lymphoma development is HIV-associated immunosuppression [48]. Loss of immune control can enable reactivation of latent oncogenic viruses. Indeed HIV-associated lymphomas are often positive for oncogenic EBV [13] or HHV-8 [14] infection, both of which have direct roles in lymphoma development. The possibility that HIV-1 Tg mice develop deficiencies in immune cells that target tumors has yet to be investigated. HIV Tg mice were negative for Abelson murine leukemia virus; however, the transgene might be activating a murine oncovirus [49] that could contribute to lymphoma.

## Conclusions

HIV Tg26 lymphomas bear many similarities to that of lymphomas occurring in HIV^+^ patients. Currently there are no murine models available for HIV-associated B cell lymphoma. The HIV Tg26 mouse model allows for the characterization of different stages of disease progression, providing a potentially valuable model for lymphomagenesis in HIV disease and for identifying potential roles of HIV-1 in B cell transformation in infected people.

## Methods

### Tg mice and generation of the HIV Tg mice colony developing lymphoma

The transgenic mouse line TgN (pNL43d14)26Lab (Tg26), was described previously [20]. The transgene contains a 3 kb deletion spanning *gag* and *pol* genes. Heterozygous mice were used for these studies since homozygotes rarely survive to weaning [22]. The colony developing lymphoma was generated by cross-breeding heterozygous Tg26 mice with skin lesions.

### Total RNA purification and real time RT-PCR

Total RNA was purified from lymphoid organs of mice. Harvested tissues were preserved in RNAlater (Qiagen), homogenized and total RNA purified using TRIzol (Invitrogen, Carlsbad, CA) and QIAamp Rneasy Mini Kit (Qiagen, Valencia, CA), and DNase I-digested (Invitrogen). cDNA synthesis was performed using iScript™ cDNA Synthesis Kit (Bio-Rad). Semiquantitative real-time RT-PCR was performed using iQ™ SYBR® Green Supermix Kit (Bio-Rad) with an ABI sequence detection system (ABI PRISM 5700). All reactions were in triplicate. Semiquantitative RT-PCR was performed to detect expression of HIV mRNAs [50] and cellular oncogene mRNAs (Additional file 1: Table S2).

### Western blot

Lymphoid organs from Tg mice were homogenized in Ripa buffer containing a protease inhibitor mixture (Sigma-Aldrich). Proteins (30 μg) were electrophoresed (12% SDS-polyacrylamide), transferred to a PVDF membrane (Bio-Rad), probed with anti-HIV-1 gp120 (Aalto Bio Reagents Ltd., Ireland), anti-HIV-1 p17 (Abnova), HIV-1 Nef Antiserum (NIH AIDS Research and Reference Reagent Program), anti-beta actin (Cell Signaling) and anti-mouse serum albumin (Abcam), incubated with respective HRP-conjugated secondary antibodies (Santa Cruz, Inc., CA), developed using an ECL chemiluminescent substrate kit (Invitrogen) and exposed to Kodak X-ray film.

### Histological analysis

Mice were anesthetized with metofane and tissues fixed overnight in 4% paraformaldehyde. Sections of paraffin-embedded tissues were cut at 5-mm thickness and stained with hematoxylin and eosin (H&E).

### Adoptive transfer

Splenocytes from a mouse with advanced spontaneous lymphoma were isolated with a tissue grinder (VWR International). CD3^+^ cells and CD3^-^ cells were separated using MACS system (Miltenyi Biotec); next CD19^+^, B220^+^ and CD117^+^ cells were isolated with magnetic bead separation. Purity of B cell populations was evaluated by flow cytometry. One million CD3^+^, CD3^-^, CD19^+^, B220^+^ and CD117^+^ cells were injected I.P. in NOD/SCID mice. Derived tumors were dissociated to single cells and analyzed for expression of lymphoid markers. All animal experiments were approved by the IACUC at UMAB.

### Flow cytometry

Single cell suspensions were analyzed by flow cytometry. The following antibodies were used (from eBioscience and BD): antibody to B220 (anti-B220; RA3-6B2), anti-CD3ϵ (17A2), anti-CD4 (GK1.5), anti-CD8a (53–6.7), anti-CD11b (anti-Mac1; M1/70), anti-CD14 (Sa2-8), anti-CD19 (1D3), anti-CD20 (AISB12), anti-CD22 (2D6), anti-CD34 (RAM34), anti-CD27 (LG.7F9), anti-CD43 (eBioR2/60), anti-CD93 (AA4.1), anti-CD117 (anti-c-Kit; 2B8), anti-CD184 (anti-CXCR4; 2B11), anti-IgD (11-26c), anti-IgG1 (A85-1), anti-IgM (II/41), anti-Sca1 (anti-Ly6A/E), anti-pre-BCR (SL-156) and anti CD127 (A7R34). Samples were acquired and analyzed with a FACSCalibur (Becton Dickinson) and FlowJo software (Tree Star, Ashland, OR), respectively.

### Immunoglobulin gene rearrangement

Ig gene rearrangements for clonal B-cell determination of mouse tumors were detected by PCR using 0.1 mg of DNA extracted from spleens of asymptomatic Tg26, and transgenic mouse tumors using the DNeasy Tissue kit (Qiagen). Primers for immunoglobulin D-Jβ gene rearrangements of the IgH gene were previously described [30]. Amplified DNA products were loaded on 6% TBE-urea gel, electrophoresed, and stained with ethidium bromide.

### Cytokine assays

Peripheral blood was collected from the eye, and plasma was separated using plasma separators (BD Microtainer). Plasma cytokines were determined using Bio-Plex Pro assay cytokine kits (Biorad) following the manufacturer’s instruction. Data from the reaction were acquired using a Luminex-based reader (Biorad).

### Statistical analysis

Analyses were performed using GraphPad Prism v 5.0. For categorical independent variables, differences between groups were assessed by using Student t test.

## Abbreviations

Ab-MLV: Abelson murine leukemia virus; HIV-1: Human Immunodeficiency Virus, Type I; HAL: HIV associated Lymphomas; HHV-8: Human herpesvirus 8; NHL: Non-Hodgkins Lymphoma; Tg: Transgenic; Tg26: Transgenic mouse line TgN (pNL43d14)26Lab.

## Competing interests

The authors declare that do not have any financial or non-financial competing interests.

## Authors’ contributions

CS designed and performed experiments, analyzed data, and wrote the manuscript; SK performed experiments and contributed to the writing of the manuscript; MR contributed to the writing of the manuscript; LI. Y performed experiments; MLK designed and performed experiments; AG. D designed and performed experiments; D.Z. designed and performed experiments and contributed to the writing of the manuscript; RG provided logistic and budget support and contributed to the writing of the manuscript; and JB supervised research, performed experiments, provided logistic and budget support, and contributed to the writing of the manuscript. All authors read and approved the final manuscript.

## Supplementary Material

Additional file 1: Figure 1S Splenic peripheral B cell population in HIV Tg mice. Splenic peripheral B cell population in four HIV Tg mice in advanced stage of splenomegaly. Flow cytometry analysis displayed: **(A)** two major populations at SSC/FSChi; **(B)** increase of CD19+ B cells; **(C)** increase of precursors B cells (B220+CD43+CD93+). **Figure 2S.** Analysis of D-J rearrangement of the IgH gene. Genomic DNAs from splenocytes isolated from HIV Tg mice progressing to splenomegaly **(A)** or at the last stage of splenomegaly **(B)** were analyzed for D-J rearrangement of the IgH gene. Splenocytes from a wild type FVBN mouse were used as a control for polyclonal B cells. Four bands corresponding to D-JH1, D-JH2, D-JH3 and D-JH4 were observed in the polyclonal B cells control. **Figure 3S.** Oncogenes expressed in the spleen of HIV tg mice. Total RNA was isolated from spleen of mice at different stage of splenomegaly and was analyzed using primers specific for oncogenes associated to lymphoma (Additional 1: Table S3) by SYBR Green semiquantitative real-time RT-PCR as described in material and methods. The fold change in each oncogene mRNA compared to the wild type FVBN control is shown relative to the change in the expression of β-actin that was measured as an internal control. The expression of each oncogene in the wild type FVBN control was set to 1. Each mouse is designated with M followed by a number indicating the percentage of total activated cells and the T/B ratio in parenthesis. Mouse number and T/B ratio are showed at the top of each graphic. Oncogenes are indicated with letters a-j where **(a)** bcl2, **(b)** bcl3, **(c)** bcl6, **(d)** c-rel, **(e)** mum1, (f) c-myc, (g) cyclin D1, (h) syk, (i) k-ras, and (j) abl. **Figure 4S.** Oncogenes expressed in the lymph node of HIV tg mice. Total RNA was isolated from lymph node of mice at different stage of splenomegaly and was analyzed using primers specific for oncogenes associated to lymphoma (Additional 1: Table S2) by SYBR Green semiquantitative real-time RT-PCR as described in material and methods. The results analysis in the lymph nodes are represented similar to the results of oncogene analysis in the spleen shown in Additional 1: Figure S3. **Figure 5S.** Percentage of mice that developed lymphoma. Six groups of NOD/SCID mice where collected for the tumorigenic experiment. Each group consisting of six mice was injected intraperitoneal with 106 B220+, CD19+, CD93+ and CD117+ splenocytes. Mice injected with CD3- and CD3+ splenocytes where used as positive and negative control, respectively. At 6 weeks after injection all CD3- injected mice become visibly ill, while all the CD3+ injected control mice remained healthy. As with HIV Tg mice, signs of disease included splenomegaly, lymphadenopathy and extra nodal enlargements in the liver and the gastrointestinal tract. **Figure 6S.** Infiltration of tumorigenic cells in lymphoid organs of NOD/SCID mice. Infiltration of CD19+ cells in lymphoid organs of NOD/SCID mice injected with splenocytes from HIV Tg mouse #28. Liver, lymph node, spleen and thymus were removed from NOD/SCID mice and single cells from each organ were stained and analyzed for CD19 and CD3 markers. Each organ contained two major populations at SSC/FSChi, corresponding to non-activated and activated populations (left panel). Flow cytometry analysis displayed CD19+ cells in the activated population of all organs analyzed (right dot plot). The dot plots shown are from one mouse and are representative of results from four different mice. **Figure 7S.** Phenotypic characterization of tumors. Flow analysis was performed in tumors from mice from groups injected with CD19+, B220+, CD93+, 30% of the control CD3-, and 70% of the control CD3-. Mice shown in figure are representative from each group. Tumors were analyzed for the following cell markers consisting in: CD3, CD19, CD43, CD93, B220, IgM, CD21, CD5, pre-BCR, Sca1, CD127, CD138, and CD34. “g1”and “g2” indicate the not activated and activated gates, respectively. **Table S1.** T/B cell ratio and precursors B cell population in HIV Tg mice. Flow analysis was performed in blood, spleen and bone marrow from wild type mouse FVBN (WT), Tg without skin lesions (TG), Tg at early stage of splenomegaly (pre-L) and a Tg at advanced stage of splenomegaly ( L). Surface expression of CD19, CD3, B220, CD43, CD93 and CD127 were reported as percentage of positive cells. A representative of four different WT, TG, pre-L and L mice is shown. **Table S2.** List of oncogenes and respective primers analyzed in this study. Additional 1: Table S3. Oligonucleotide primers for detecting v-abl from A-MuLV genome [GenBank accession AF033812]. Click here for file
