# Supplementary material for: B cell lymphoma in hiv transgenic mice
Source: Retrovirology. 2013 Aug 28;10:92. doi: 10.1186/1742-4690-10-92 (PMC3847158; doi:10.1186/1742-4690-10-92)
Supplement: Additional file 1: Figure 1S — Splenic peripheral B cell population in HIV Tg mice. Splenic peripheral B cell population in four HIV Tg mice in advanced stage of splenomegaly. Flow cytometry analysis displayed: (A) two major populations at SSC/FSChi; (B) increase of CD19+ B cells; (C) increase of precursors B cells (B220+CD43+CD93+). Figure 2S. Analysis of D-J rearrangement of the IgH gene. Genomic DNAs from splenocytes isolated from HIV Tg mice progressing to splenomegaly (A) or at the last stage of splenomegaly (B) were analyzed for D-J rearrangement of the IgH gene. Splenocytes from a wild type FVBN mouse were used as a control for polyclonal B cells. Four bands corresponding to D-JH1, D-JH2, D-JH3 and D-JH4 were observed in the polyclonal B cells control. Figure 3S. Oncogenes expressed in the spleen of HIV tg mice. Total RNA was isolated from spleen of mice at different stage of splenomegaly and was analyzed using primers specific for oncogenes associated to lymphoma (Additional 1: Table S3) by SYBR Green semiquantitative real-time RT-PCR as described in material and methods. The fold change in each oncogene mRNA compared to the wild type FVBN control is shown relative to the change in the expression of β-actin that was measured as an internal control. The expression of each oncogene in the wild type FVBN control was set to 1. Each mouse is designated with M followed by a number indicating the percentage of total activated cells and the T/B ratio in parenthesis. Mouse number and T/B ratio are showed at the top of each graphic. Oncogenes are indicated with letters a-j where (a) bcl2, (b) bcl3, (c) bcl6, (d) c-rel, (e) mum1, (f) c-myc, (g) cyclin D1, (h) syk, (i) k-ras, and (j) abl. Figure 4S. Oncogenes expressed in the lymph node of HIV tg mice. Total RNA was isolated from lymph node of mice at different stage of splenomegaly and was analyzed using primers specific for oncogenes associated to lymphoma (Additional 1: Table S2) by SYBR Green semiquantitative real-time RT-PCR as described in mate [file 1742-4690-10-92-S1.pdf]

ADDITIONAL DATA FROM PAPER:

B CELL LYMPHOMA IN HIV TRANSGENIC MICE

[1]Sabrina Curreli, [1]Selvi Krishnan, [1]Marvin Reitz, [1]Lunardi-Iskandar Y., [1,4]Lafferty M., [1,4]Garzino-Demo A., [1,3]Davide Zella, [1,2]Gallo Robert C., and [1]Joseph Bryant.

[1]Institute of Human Virology and [2]Department of Medicine, [3]Biochemistry and Molecular Biology and [4]Microbiology and Immunology, University of Maryland School of Medicine, Baltimore, MD 21201.

Figure 1S

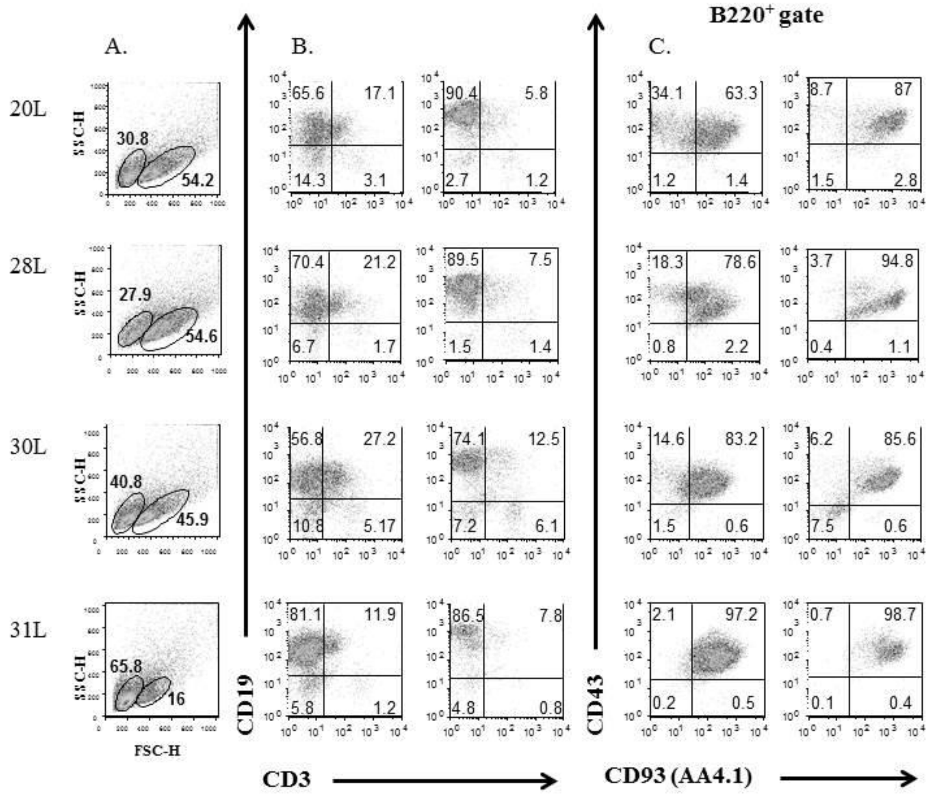

Figure 1S. Splenic peripheral B cell population in HIV Tg mice. Splenic peripheral B cell population in four HIV Tg mice in advanced stage of splenomegaly. Flow cytometry analysis displayed: (A) two major populations at SSC/FSC_{hi}; (B) increase of $CD19^{+}$ B cells; (C) increase of precursors B cells ($B220^{+}CD43^{+}CD93^{+}$).

Figure 2S

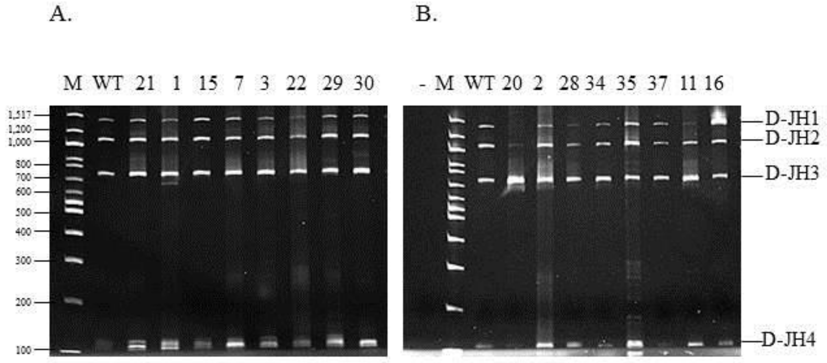

Figure 2S. Analysis of D-J rearrangement of the IgH gene. Genomic DNAs from splenocytes isolated from HIV Tg mice progressing to splenomegaly (A) or at the last stage of splenomegaly (B) were analyzed for D-J rearrangement of the IgH gene. Splenocytes from a wild type FVBN mouse were used as a control for polyclonal B cells. Four bands corresponding to D-JH1, D-JH2, D-JH3 and D-JH4 were observed in the polyclonal B cells control.

Figure 3S

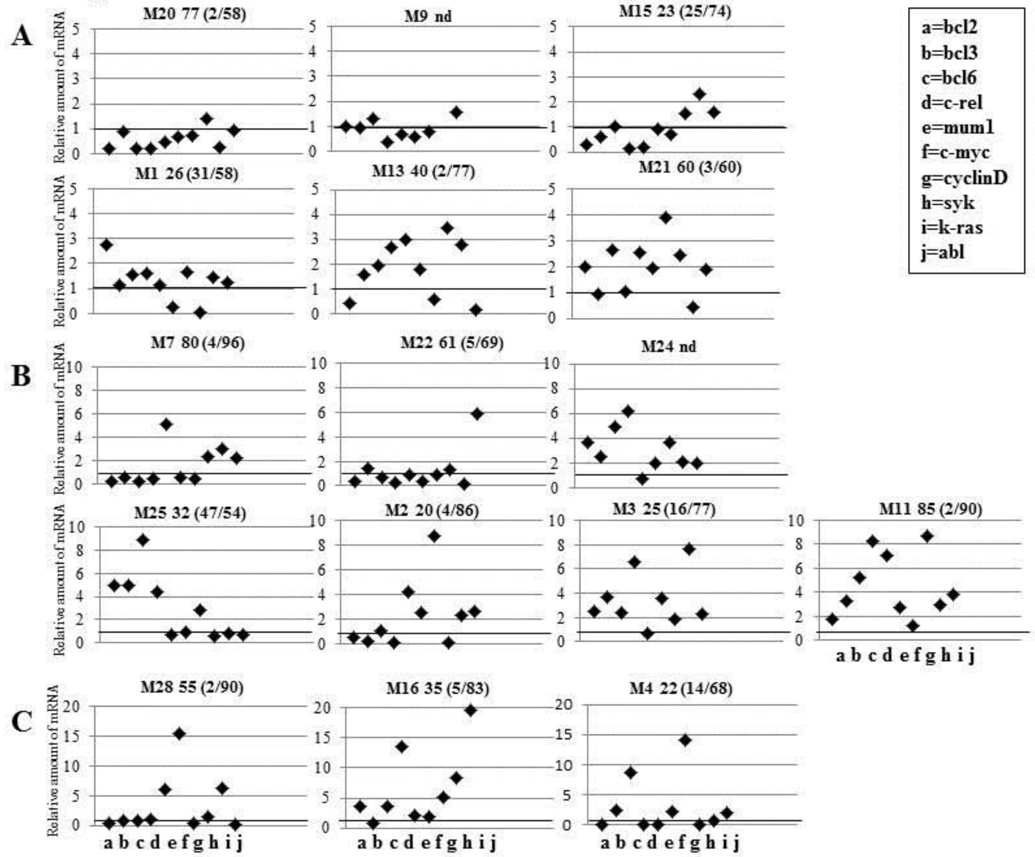

Figure 3S. Oncogenes expressed in the spleen of HIV tg mice. Total RNA was isolated from spleen of mice at different stage of splenomegaly and was analyzed using primers specific for oncogenes associated to lymphoma (Table 3S) by SYBR Green semiquantitative real-time RT-PCR as described in material and methods. The fold change in each oncogene mRNA compared to the wild type FVBN control is shown relative to the change in the expression of β-actin that was measured as an internal control. The expression of each oncogene in the wild type FVBN control was set to 1. Each mouse is designated with M followed by a number indicating the percentage of total activated cells and the T/B ratio in parenthesis. Mouse number and T/B ratio are showed at the top of each graphic. Oncogenes are indicated with letters a-j where (a) bcl2, (b) bcl3, (c) bcl6, (d) c-rel, (e) mum1, (f) c-myc, (g) cyclin D1, (h) syk, (i) k-ras, and (j) abl.

Figure 4S

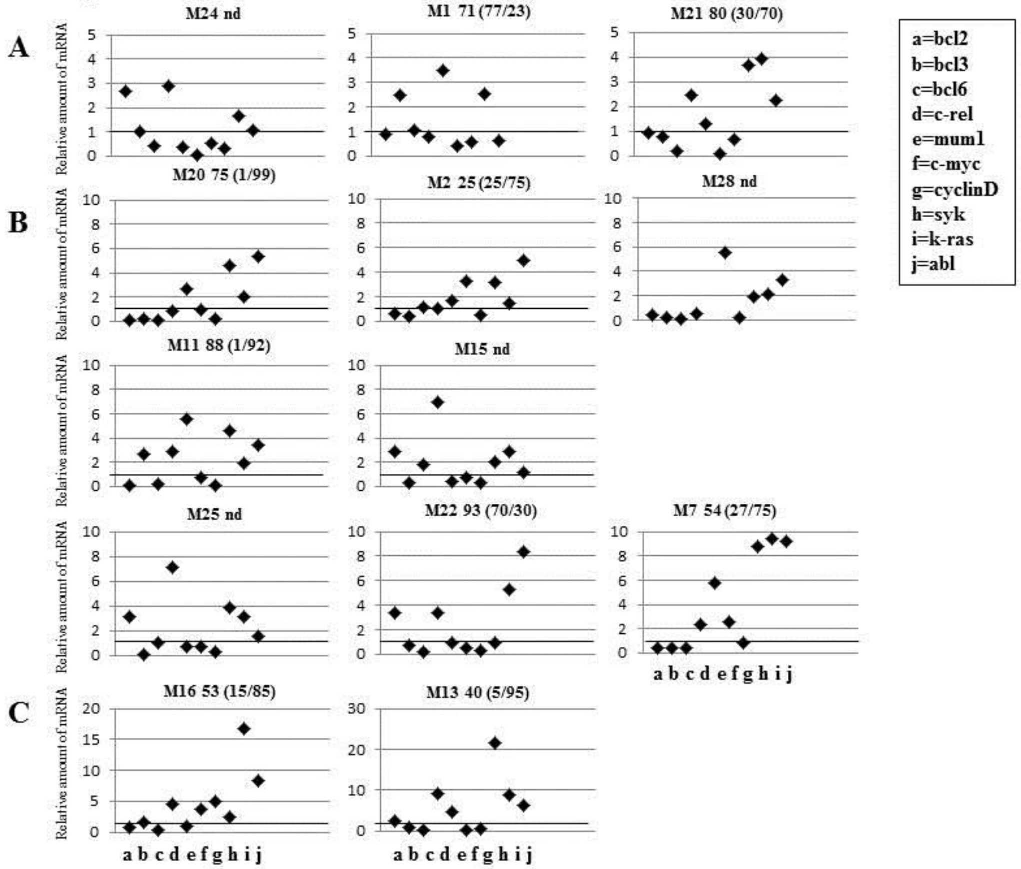

Figure 4S. Oncogenes expressed in the lymph node of HIV tg mice. Total RNA was isolated from lymph node of mice at different stage of splenomegaly and was analyzed using primers specific for oncogenes associated to lymphoma (Table 2S) by SYBR Green semiquantitative real-time RT-PCR as described in material and methods. The results analysis in the lymph nodes are represented similar to the results of oncogene analysis in the spleen shown in Figure 3S.

Figure 5S

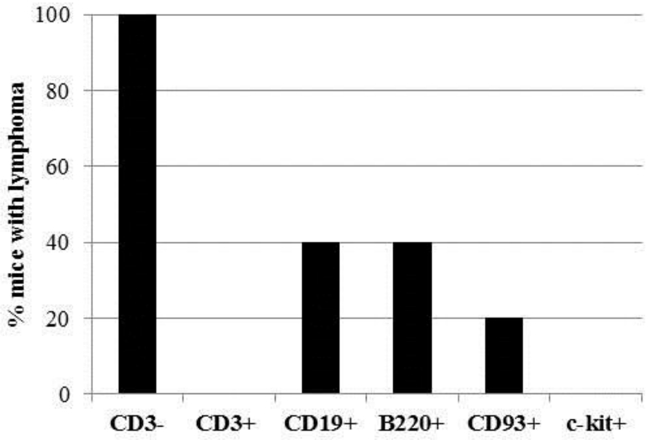

Figure 5S. **Percentage of mice that developed lymphoma**. Six groups of NOD/SCID mice where collected for the tumorigenic experiment. Each group consisting of six mice was injected intraperitoneal with 10^6 $B220^+$, $CD19^+$, $CD93^+$ and $CD117^+$ splenocytes. Mice injected with $CD3^-$ and $CD3^+$ splenocytes where used as positive and negative control, respectively. At 6 weeks after injection all $CD3^-$ injected mice become visibly ill, while all the $CD3^+$ injected control mice remained healthy. As with HIV Tg mice, signs of disease included splenomegaly, lymphadenopathy and extra nodal enlargements in the liver and the gastrointestinal tract.

Figure 6S

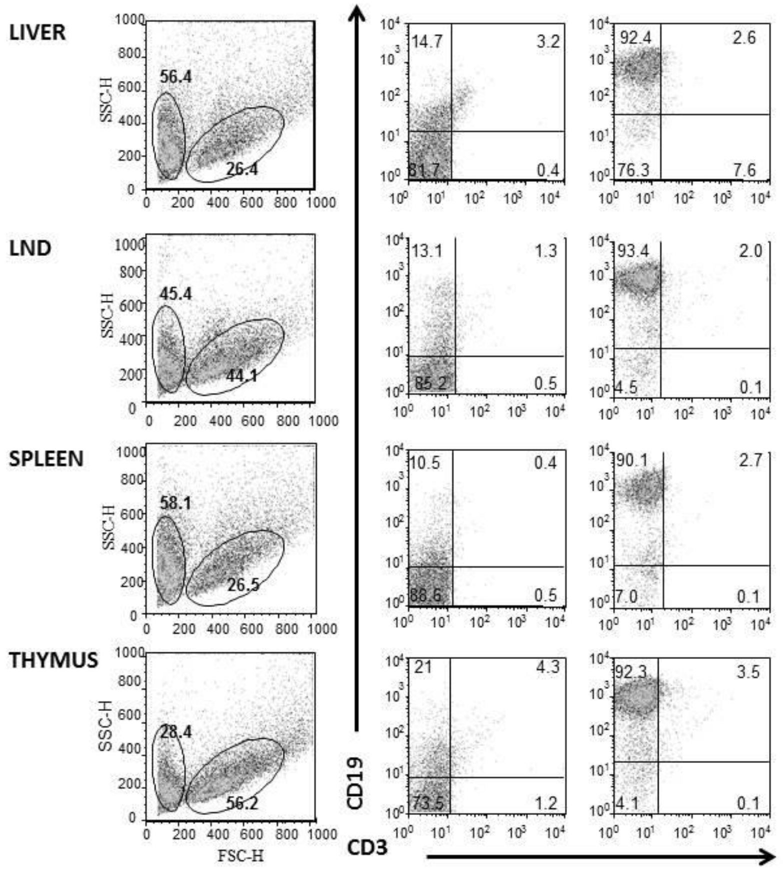

Figure 6S. Infiltration of tumorigenic cells in lymphoid organs of NOD/SCID mice. Infiltration of $CD19^+$ cells in lymphoid organs of NOD/SCID mice injected with splenocytes from HIV Tg mouse #28. Liver, lymph node, spleen and thymus were removed from NOD/SCID mice and single cells from each organ were stained and analyzed for CD19 and CD3 markers. Each organ contained two major populations at SSC/FSC_{hi}, corresponding to non-activated and activated populations (left panel). Flow cytometry analysis displayed $CD19^+$ cells in the activated population of all organs analyzed (right dot plot). The dot plots shown are from one mouse and are representative of results from four different mice.

Figure 7S

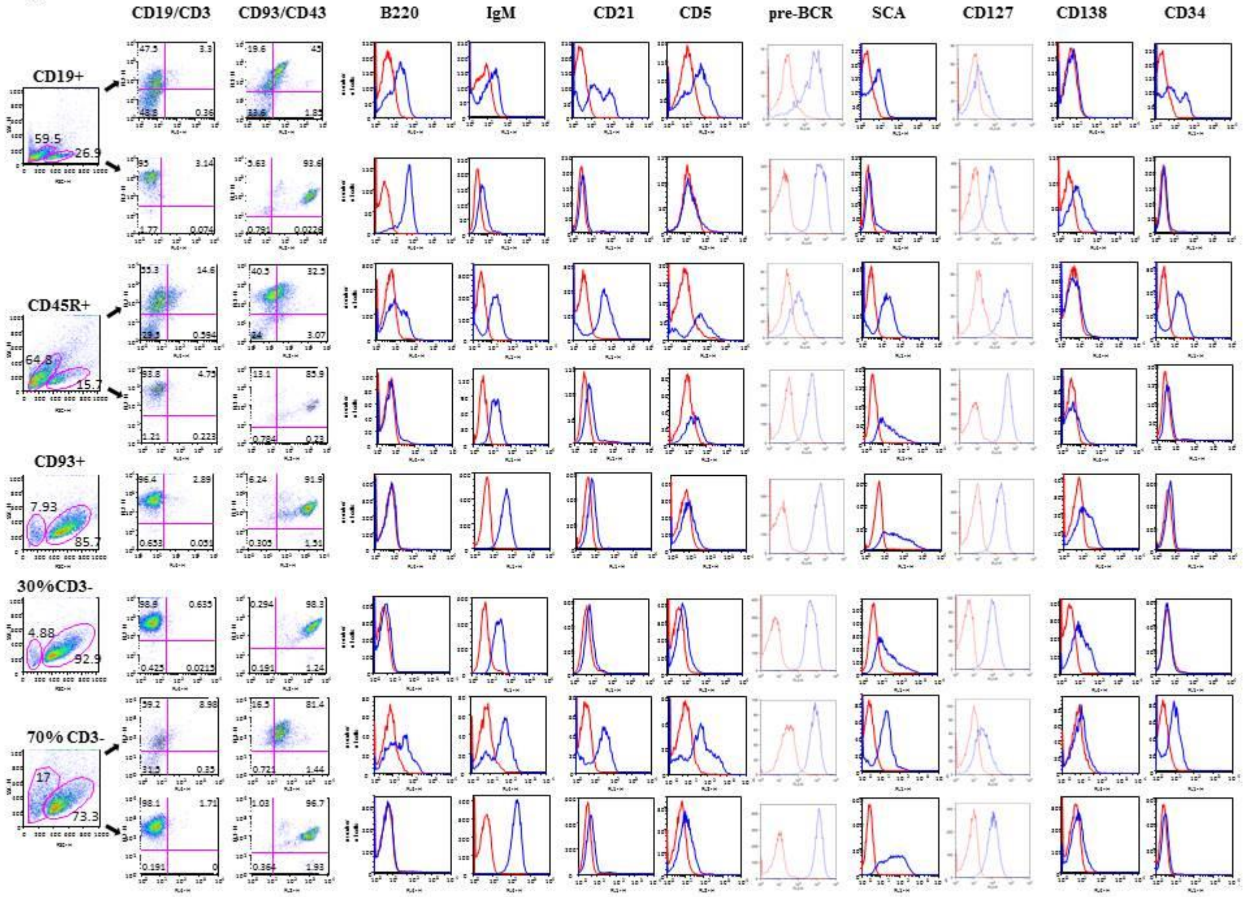

Figure 7S. Phenotypic characterization of tumors. Flow analysis was performed in tumors from mice from groups injected with $CD19^+$, $B220^+$, $CD93^+$, 30% of the control $CD3^-$, and 70% of the control $CD3^-$. Mice shown in figure are representative from each group. Tumors were analyzed for the following cell markers consisting in: CD3, CD19, CD43, CD93, B220, IgM, CD21, CD5, pre-BCR, Sca1, CD127, CD138, and CD34. “g1”and “g2” indicate the not activated and activated gates, respectively.

Table 1S. T/B cell ratio and precursors B cell population in HIV Tg mice

		WT	TG	pre-L	L
BLOOD	CD3/CD19	35/12	45/7	45/11	2.5/78.5
	$B220^{+}CD43^{+}CD93^{+}$	0.4%	8%	0.2%	68%
	$CD19^{+}CD127^{+}$	2%	1%	1%	67%
SPLEEN	CD3/CD19	23.5/27	36/52.5	13/18	1/52
	$B220^{+}CD43^{+}CD93^{+}$	0.7%	2.8%	1.6%	42.5%
	$CD19^{+}CD127^{+}$	2.5%	5.5%	1.2%	46%
BONE MARROW	CD3/CD19	0/10	1/9.5	4/11.5	1/58
	$B220^{+}CD43^{+}CD93^{+}$	8.1%	15%	9%	42%
	$CD19^{+}CD127^{+}$	5%	7%	6%	52%

Flow analysis was performed in blood, spleen and bone marrow from wild type mouse FVBN (WT), Tg without skin lesions (TG), Tg at early stage of splenomegaly (pre-L) and a Tg at advanced stage of splenomegaly (L). Surface expression of CD19, CD3, B220, CD43, CD93 and CD127 were reported as percentage of positive cells. A representative of four different WT, TG, pre-L and L mice is shown.

Table 2S. List of oncogenes and respective primers analyzed in this study

**Gene**	**GenBank™ Accession**	**Forward primer**	**sequence**	**Reverse primer**	**sequence**	**Size product**
β actin	NM_007393.3	mβF	TGCAGCTCCTTCGTTGCCGG	mβR	TCACACCCTGGTGCCTAGGGC	70bp
bcl2	NM_009741.3	bcl2F	AACGGAGGCTGGGATGCCTT	bcl2R	AGTGATGCAGGCCCCGACCA	120bp
bcl3	NM_033601	bcl3F	AACTCCTGCTGCTGCACGGC	bcl3R	CGTGCGCACCAGAGGCAGAA	104bp
bcl6	NM_009744	bcl6F	GTGAACAGGTCCCTGGCAGGC	bcl6R	GGGGACTGAGAGCCGCAGGA	101bp
cyclinD1	NM_0076631.2	cyclD1F	GGCTGCGATGCAAGGCCTGA	cyclD1R	CGGAGGCAGTCCGGGTCACA	108bp
c-myc	NM_001177352.1	c-mycF	TCCACCGCCGATCAGCTGGA	c-mycR	TGGCAGCGGCTGAGAAACCG	148bp
c-rel	NM_009044.2	c-relF	GAGCCATGGCCTCGAGTGGA	c-relR	GTCTGTGCTGCGCTCCCCTG	131bp
k-ras	NM_021284.6	krasF	AGACACGAAACAGGCTCAGGAGTT	krasR	AGAAGGCATCGTCAACACCCTGTC	95bp
mum-1	NM_013674	mumF	ATCACAGCTCATGTGGAACCTC	mumR	TGCCCTGTCAGAGTATTTCTTCTC	175bp
syk	NM_011518.2	sykF	GATCTGGCTGCGCGGAACGT	sykR	TCCCGTGGGTCTGGGCCTTG	118bp
c-abl	NM_001112703; NM_009594	ablF	CCCAGCTCCCAAGCGCAACA	ablR	CCCGTACTGGCCTCCACCCA	118bp

Table 3S. Oligonucleotide primers for detecting v-abl from A-MuLV genome [GenBank accession AF033812]

**F primer**	**Sequence**	**R primer**	**Sequence**	**Size product**
124F	CCGTCAGCGGGGGTCTTTCA	124R	TACCTCCCGGTGGTGGGTCG	84bp
310F	GCGGACCCGTGGTGGAACTG	310R	AAACGGCCCCCGAAGTCCCT	81bp
382F	GGGCCGTTTTTGTGGCCCGA	382R	CAGACGGAGGCGGGAACTGT	132bp
4664F	ACGCCATTTTGCAAGGCATGGA	4664R	GCCGGGGCAGGAACTGCTTA	129bp
